# Sickle Cell Disease: Genetics, Cellular and Molecular Mechanisms, and Therapies

**DOI:** 10.1155/2012/143594

**Published:** 2012-08-22

**Authors:** Betty S. Pace, Solomon F. Ofori-Acquah, Kenneth R. Peterson

**Affiliations:** ^1^Department of Pediatrics, Georgia Health Sciences University, 1120 15th Street, BT-1852, Augusta, GA 30912, USA; ^2^Department of Pediatrics, Emory University School of Medicine, 2015 Uppergate Drive NE, Atlanta, GA 30322, USA; ^3^Department of Biochemistry and Molecular Biology, University of Kansas Medical Center, 3901 Rainbow Boulevard, MS 3030, Kansas City, KS 66160, USA

Sickle cell disease (SCD) is a global public health disorder that affects millions of people across the globe. It is a monogenic disorder caused by an A-to-T point mutation in the *β*-globin gene that produces abnormal hemoglobin S (Hb S), which polymerizes in the deoxygenated state, resulting in physical deformation or sickling of erythrocytes. Sickle erythrocytes promote vaso-occlusion and hemolysis, which are two major cellular hallmarks of the disease. Rapid advances made in understanding the molecular genetics of SCD in the early part of the 20th century have not been matched by comparable progress towards understanding its clinical complications, and developing effective therapies. Contemporary reevaluation of SCD as the product of multiple gene interactions promises to overcome the historical constraints of the single-gene disease paradigm that has inevitably impeded translation of research discoveries into clinical benefit.

Two landmark papers in the late 1940s by the Nobel laureates Linus Pauling and Janet Watson provided the molecular bases for SCD and a rational strategy to treat the disease. The publication by Pauling et al., *Sickle Cell Anemia, a Molecular Disease*, in *Nature* in 1949 established SCD as the first molecular human disease, and it established the inheritance pattern of the disorder and of monogenic diseases generally. In addition, that seminal work laid the foundation for the explosion of knowledge in human molecular genetics decades later that gave birth to a new discipline called *gene therapy*. The publication by Watson *The Significance of the Paucity of Sickle Cells in Negro Infants* provided the concept that fetal hemoglobin (Hb F) ameliorates the clinical presentation of SCD for the first time in 1948, ushering in one of the most intensely studied areas of SCD research. 

Advancements in *Genetics, Cellular and Molecular Mechanisms, and Therapy of SCD* in the two decades following the seminal works by Pauling and Watson were driven primarily by studies on the erythrocyte, involving polymerization of Hb S and antisickling hemoglobin variants, rheology, and red cell membrane. A highlight amongst these studies was the landmark work by Kan and Dozy published in the article *DNA Sequence Adjacent to the Human Beta-Globin Structural Gene: Relationship to Sickle Mutation. *That study described the presence of single-nucleotide polymorphisms in the human genome for the first time, and it initiated a new avenue of research that led to the discovery of the multicentric origin of the sickle mutation, and it laid the foundations for genetic association studies in SCD, which are currently a major focus of several investigations. 

The scope of SCD research expanded beyond the erythrocyte in the 1980s to encompass vascular biology, notably the endothelium, coagulation, and inflammation. Twenty years later, the most compelling evidence that these factors play a critical role in the pathogenesis of SCD is the demonstration that tumor necrosis factor induced adhesion of leukocytes to the vascular endothelium provides the initial cellular events of vaso-occlusion in a mouse model of SCD. Paradigm-shifting insights into the mechanisms of globin gene expression spearheaded by the discovery of the locus control region (LCR) by two groups in the 1990s heralded a new era in SCD research. First, these insights helped to create developmentally regulated and clinically relevant transgenic mouse models of SCD. Second, they permitted the development of efficacious DNA vectors for gene therapy of SCD that continue to improve as novel elements of gene delivery systems become available and are incorporated into newer generation vectors.

The current special issue of *Anemia* contains original research articles about progress made towards Hb F induction using novel pharmacological agents and artificial zinc finger transcription factors, and a web-based tool to evaluate adherence to hydroxyurea therapy. The latter tool represents efforts to integrate new technology to improve the clinical care of individuals with SCD. Also included in this issue is the first report from a Congolese group of the association of Hb F levels with clinical severity in this population. Several articles report alteration in redox environment and link this phenomenon to impaired hematopoietic progenitor and stem cell function improved by treatment with n-acetyl cysteine in transgenic SCD mice, reduced migration of endothelial progenitors cells derived from children who have SCD, and lastly an association of oxidative stress markers with an atherogenic index in adults with sickle nephropathy. What is known about the deleterious effects of sickling on the genitourinary tract and the role of cyclic nucleotide signaling is reviewed. Finally, articles report two endothelial dysfunction including increased activity of the elastase cathepsin K, and age-dependent increase, in vascular permeability, that culminates in pulmonary edema in middle-aged SCD mice. 

The wide variety of experimental studies in this special issue represents potentially new therapeutic tools, ranging from novel approaches for Hb F induction, improved stem cell function and a biomarker to predict risk for SCD nephropathy. Furthermore, the findings of endothelial dysfunction via upregulated cathepsin activity may represent a new pharmacologic target to block accelerated arterial disease observed in children with SCD. The reports in this issue will aid research efforts to close the gap between understanding SCD genetics and developing effective new clinical care approaches and therapeutic options.

*Betty S. Pace* *Solomon F. Ofori-Acquah* *Kenneth R. Peterson*

